# Books and Magazines

**Published:** 1907-06

**Authors:** 


### Books and Magazines.

Progressive Medicine, Vol. I, March, 1907. A Quarterly Digest of Advances, Discoveries and Improvements in the Medical and Surgical Sciences. Edited by Hobart Amory Hare, M. D., Professor of Therapeutics and Materia Medica in the Jefferson Medical College of Philadelphia. Octavo, 280 pages, with illustrations. Per annum, in four cloth-bound volumes, $9; in paper binding, $6, carriage paid to any address. Lea Brothers & Co., Publishers, Philadelphia and New York.

The March issue of Progressive Medicine (Volume 1, of the series of 1907) crystallizes the experience of a host of skilled observers, in all parts of the world, during the past year. In the opening pages Dr. Charles H. Frazier reviews the current literature of the Surgery of the Head, Neck and Thorax. The practical bearing of edema of the brain, in consideration of early operations for intracranial injuries, is carefully detailed. Under "Traumatic Intracranial Hemorrhage," he says:

"It has been shown by autopsy and operation that cerebral contusion is not as frequent a complication of *subdural hemorrhage* as once supposed, and it should be remembered in the first place, that cerebral contusion is by no means a contra-indication to operation and may be favorably affected by the removal of a large clot."

A *résumé* of 530 pages of Fractures of the Base of the Skull; Crile's original methods of controlling hemorrhage during operations upon the head; the technique of palliative operations; cerebral abscess; operations on the pituitary body; cerebellar surgery; epilepsy; trifacial neuralgia; paraffin prosthesis; Ludwig's angina; the results of thyroidectomy in over a thousand cases; an improved technique for amputation of the breast; and a summary of sixteen recent cases of suture of the heart are some of the matters discussed.

---

The Essentials of Histology, Descriptive and Practical.—For the use of Students. By Edward A. Schäfer, F. R. S., Professor of Physiology in University College, London. New (7th) edition, revised and enlarged. Octavo, 507 pages, with 552 illustrations. Cloth, $3.50, net. Lea Brothers & Co., Philadelphia and New York, 1907.

A book which achieves seven editions with constantly increasing growth in itself and broadening acceptance on the part of its public, possesses indubitable and proved merit. Especially is this true in so highly competitive a department of medical literature as histology. The survival of the fittest is as true of books as of animals, and conversely the proof of fitness is survival. The reasons for the favor merited by Professor Schäfer's Essentials of Histology are evident. It gives a well arranged course covering the minute structure of every human tissue, and it does this in such clear language and with such a wealth of effective engravings that the difficulties of both student and teacher are minimized. It is perhaps the most richly illustrated book in the field. A feature of special value new in this edition is the abundant use of colors. This thin volume is really a fairly large and full text-book, for it contains 500 pages, but it is printed on exceptionally fine paper for portability. It is an authoritative and favorite text-book, again revised to the latest date, and attractively presented in every detail.

---

A Treatise on the Practice of Medicine.—For Practitioners and Students. By Arthur R. Edwards, M. D., Professor of the Principles and Practice of Medicine and Clinical Medicine in the Northwestern University Medical School, Chicago. Octavo, 1328 pages, with 101 engravings and 19 plates. Cloth, $5.50, net; leather, $6.50, net. Lea Brothers & Co., Philadelphia and New York, 1907.

A new work in so broad and well tilled a field as the literature of practice may be expected to present its credentials. In the case of Professor Edwards' book they are of such character as to form presumptive evidence of its value and to give it immediate prestige. The author has for years occupied one of the most important chairs in the country, and his skill in filling it is manifested by his steadily large classes. Teaching is excellent training for the teacher himself. It enforces two essential points, perspective and clearness. Without either a large subject is befogged. The successful teacher must know how to present a picture to the mind with due emphasis on what is important, and with every item in its relative position and coloring, all being in clear and definite language. He must know his subject and speak with authority. Possessing these qualifications he will write a well-balanced book and save time and energy for teacher, student and practitioner alike. This our author has accomplished for all classes of readers. His book is well rounded, covering theory as leading up to and explaining facts, and never forgetting that the aim of medicine is application. Hence the practitioner will find guidance in understanding his cases and unusually full advice in their treatment, including abundant prescriptions accepted as best at the present day.

